# Efficacy of implementation strategies of an evidenced-based awakening and breathing protocol

**DOI:** 10.1186/cc9583

**Published:** 2011-03-11

**Authors:** O Almuslim, M Rezk, N Hassan

**Affiliations:** 1 King Fahad Specialist Hospital - Dammam, Saudi Arabia

## Introduction

A protocol that paired spontaneous awakening trials (SAT) and spontaneous breathing trials (SBT) decreased duration of mechanical ventilation (DMV), ICU length of stay (LOS) and mortality [1]. We studied the efficacy of multifaceted implementation strategies (MIS) of an evidenced-based protocol at a tertiary academic center.

## Methods

This was a prospective observational cohort study with historical control. The cohort consisted of consecutive patients who were extubated at least once during the ICU stay. The intervention was MIS of a quality improvement (QI) protocol pairing SAT and SBT. These strategies included: preprinted daily order sheets, structured daily multidisciplinary rounds, QI monitoring and regular feedback to the ICU staff. The outcomes: DMV, ICU LOS, reintubation and hospital mortality. Chi-square and *t *tests, adjusted logistic and Cox regressions were used.

## Results

**Figure 1:**
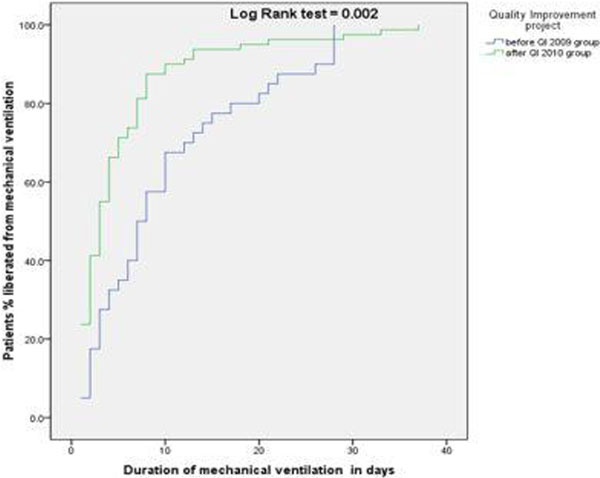
**Time to extubation KM curve**.

Total patients *n *= 120 (2009, *n *= 40; 2010, *n *= 80). The baseline characteristics were imbalance for age and APACHE II. The 2010 group (after QI) had less DMV, ICU LOS and hospital mortality (Table 1). The adjusted hazard ratio in reducing time to extubation = 0.57 (95% CI = 0.37 to 0.88) and adjusted odds ratio for hospital mortality = 0.27 (95% CI = 0.12 to 0.67) in the 2010 group. See Figure 1.

**Table 1:** Main outcomes

	2009 group (*n *= 40)	2010 group (*n *= 80)	*P *value
MV duration (days)	10.3 (SD 8.6)	5.3 (SD 6.7)	< 0.01
ICU LOS (days)	12.4 (SD 8.3)	8.6 (SD 9.1)	0.03
Reintubation	33% (*n *= 13)	18% (*n *= 14)	0.06
Hospital mortality	60% (*n *= 24)	20% (*n *= 16)	< 0.01

## Conclusions

MIS of a paired SAT and SBT protocol reduced duration of MV, ICU LOS and hospital mortality.
